# The Purinergic Landscape of Type 2 Diabetes Mellitus

**DOI:** 10.3390/molecules27061838

**Published:** 2022-03-11

**Authors:** Rocio Edith Garcia-Jacobo, Leticia Scussel Bergamin, Valentina Vultaggio-Poma, Maria Luiza Thorstenberg, Mario Tarantini, Mariana Haydee García-Hernández, Francesco Di Virgilio

**Affiliations:** 1 Department of Medical Sciences, University of Ferrara, 44121 Ferrara, Italy; rocioedith.garciajacobo@unife.it (R.E.G.-J.); scsltc@unife.it (L.S.B.); vltvnt@unife.it (V.V.-P.); thorstenbergml@gmail.com (M.L.T.); mario.tarantini@studenti.unipd.it (M.T.); 2 Unidad de Investigación Biomédica, Delegación Zacatecas, Instituto Mexicano del Seguro Social, IMSS, Zacatecas 98000, Mexico; mariana.garciah@imss.gob.mx

**Keywords:** extracellular ATP, P2X7 receptor, type 2 diabetes mellitus, inflammation

## Abstract

Adenosine triphosphate (ATP) is the key energy intermediate of cellular metabolic processes and a ubiquitous extracellular messenger. As an extracellular messenger, ATP acts at plasma membrane P2 receptors (P2Rs). The levels of extracellular ATP (eATP) are set by both passive and active release mechanisms and degradation processes. Under physiological conditions, eATP concentration is in the low nanomolar range but can rise to tens or even hundreds of micromoles/L at inflammatory sites. A dysregulated eATP homeostasis is a pathogenic factor in several chronic inflammatory diseases, including type 2 diabetes mellitus (T2DM). T2DM is characterized by peripheral insulin resistance and impairment of insulin production from pancreatic β-cells in a landscape of systemic inflammation. Although various hypoglycemic drugs are currently available, an effective treatment for T2DM and its complications is not available. However, counteracting systemic inflammation is anticipated to be beneficial. The postulated eATP increase in T2DM is understood to be a driver of inflammation via P2X7 receptor (P2X7R) activation and the release of inflammatory cytokines. Furthermore, P2X7R stimulation is thought to trigger apoptosis of pancreatic β-cells, thus further aggravating hyperglycemia. Targeting eATP and the P2X7R might be an appealing novel approach to T2DM therapy.

## 1. Introduction

Diabetes mellitus (DM) is a widespread metabolic disorder featuring decreased peripheral insulin sensitivity, impaired insulin secretion from pancreatic β-cells, and overall dysregulation of glucose metabolism [1,2]. In 2021, approximately 537 million adults had diabetes (https://www.idf.org/; accessed 21 January 2022), and it is anticipated that by 2045, this number will rise to 693 million, making diabetes one of the most common and fastest growing diseases worldwide [3,4]. Diabetes is conventionally divided into: (i) type 1 diabetes mellitus (T1DM; autoimmune form) which is characterized by increased blood levels of autoantibodies directed against insulin-producing β-cells, and (ii) type 2 diabetes mellitus (T2DM; non-autoimmune form) which is characterized by increased peripheral resistance to insulin and decreased insulin secretion by pancreatic β-cells [3,5,6]. T2DM is a multifactorial disease caused by genetic and environmental factors, mainly obesity and dyslipidemia [3,5,6]. In addition to the well-established role in fat storage and lipid metabolism regulation, adipose tissue is also endowed with immune functions [7,8]. Adipocytes produce adiponectin, resistin, IL-6, TNF-α, MCP-1, and IL-1β [7]. ecently, a correlation has been suggested between proinflammatory cytokines secreted by adipose tissue and T1DM [7]. Proinflammatory cytokines, together with cytotoxic T cells, cause β-cell injury and the associated release of autoantigens and endogenous “danger signals” responsible for promoting pathologic self-antigen presentation [7]. Macrophages and other immune cells residing in the immune tissue secrete large quantities of proinflammatory cytokines exacerbating inflammation and insulin resistance [9].

Some experimental models for studying DM are available; however, they all have limitations. Both large (non-human primates, pigs, dogs, and cats) and small (rabbits, rats, and mice) mammals are used. Rodents are widely used for their small body sizes, ease of handling, low dietary requirements and affordable costs. Commonly used rodent models for T2DM research include partial pancreatectomy models, alloxan/streptozotocin (STZ) models, high fat diet (HFD) or fructose feeding models, models with a combination of HFD feeding and STZ injection, models with a combination of fructose feeding and STZ injection, nicotinamide-STZ models, models with an injection of monosodium glutamate, and intrauterine growth reduction models. For T1DM research, the main rodent models used are the STZ model, immunodeficient transgenic mice expressing human gene constructs (NOD-Rag1null IL-2rgnull Ins2Akita), virus-induced transgenic models, or spontaneous models [10,11].

Main complications of DM are stroke, atherosclerosis, coronary artery disease, diabetic nephropathy, diabetic retinopathy, and peripheral diabetic neuropathy [6]. Therefore, a better understanding of its onset and progression is desirable in order to devise new and more effective therapies. The purinergic system, extracellular ATP (eATP) and P2 receptors (P2Rs), might be an appealing target.

## 2. Purinergic Signaling

ATP is the fundamental energy intermediate in all living organisms and a ubiquitous extracellular mediator of several pathophysiological processes, e.g., cardiovascular function, neurotransmission, muscle contraction, bone metabolism, glucose homeostasis, inflammation, immunity, and cancer [12,13,14]. Intracellular ATP (iATP), synthesized by glycolysis and oxidative phosphorylation, is freely present in the cell cytoplasm or stored in cytoplasmic vesicles or organelles. In pancreatic β-cells, ATP is co-stored with insulin in secretory granules [15,16]. The iATP concentration is in the millimolar range (3–10 mM) while, in physiological conditions, the eATP concentration is in the nanomolar range [13], thus setting a steep concentration gradient across the plasma membrane. However, at sites of inflammation or tissue damage, the eATP concentrations can rise to tens or even hundreds micromoles/L [17]. Release of ATP into the extracellular space can occur by passive efflux due to plasma membrane damage or by controlled release via regulated mechanisms such as vesicular exocytosis, plasma membrane-derived microvesicles, ATP binding cassette (ABC) transporters, connexins, pannexin channels, calcium homeostasis modulators (CALMH) channels, or the P2X7R itself [12]. Of note, ATP is co-secreted together with insulin in a glucose-dependent fashion [15]. As for any bona fide extracellular messenger, released eATP must be quickly degraded to terminate stimulation and prevent receptor desensitization. Hydrolysis of eATP to ADP, AMP, and adenosine is catalyzed by the sequential activity of soluble or membrane-bound ectonucleotidase families, including members of the ectonucleoside triphosphate diphosphohydrolases (E-NTPDases), ectonucleotide pyrophosphatase/phosphodiesterases (E-NPPs), ectoalkaline phosphatases (ALPs), and ecto-5′nucleotidase/CD73 [18]. The main sensors of eATP and its metabolites are P2Rs and P1Rs. P2Rs are targeted by different nucleotide ligands, i.e., ATP, ADP, UTP, UDP, and UDP-glucose, while P1Rs are ligated and activated only by adenosine. Four P1R subtypes are known: A1, A2A, A2B, and A3. All of them are metabotropic, G-protein-coupled receptors. P2Rs are divided into two subgroups: P2YRs (metabotropic, G-protein-coupled receptors) and P2XRs (ionotropic, intrinsic ion channels) [19,20,21,22]. Eight P2YR subtypes have been identified in humans (P2Y_1_R, P2Y_2_R, P2Y_4_R, P2Y_6_R, P2Y_11_R, P2Y_12_R, P2Y_13_R and P2Y_14_R, the P2Y_11_R is missing in rodents), further classified on the basis of the respective coupled G protein into: (i) P2Y_1_R, P2Y_2_R, P2Y_4_R, P2Y_6_R and P2Y_11_R, which are coupled to Gq-protein and phospholipase C activation, and (ii) P2Y_12_R, P2Y_13_R and P2Y_14_R, which are coupled to Gi and the inhibition of adenylate cyclase. The P2Y_11_R couples to both Gq and Gs, thus triggering the increase of intracellular Ca^2+^ and cAMP [21,23]. While P2YRs are activated by different nucleotides (e.g., ATP, ADP, UTP, UDP, and UDP-glucose), P2XRs are solely activated by eATP [19,20,22]. P2XRs are intrinsic ion channels that mediate Na^+^ and Ca^2+^ influx and K^+^ efflux [19,20,22,23]. Seven P2XR subtypes have been identified: P2X1R, P2X2R, P2X3R, P2X4R, P2X5R, P2X6R and P2X7R [24]. Each receptor subunit consists of a N-terminus, two transmembrane domains separated by a bulky extracellular loop, and a C-terminal tail varying in length depending upon the P2XR subtype [24,25]. Among P2X receptors, the P2X7R has unique functional and molecular properties that make it a potential therapeutic target in several pathologies [26,27].

### The P2X7 Receptor

The P2X7R, the largest in the P2XR family, is made by the assembly of three subunits (homotrimer) of 595 amino acids each and a molecular mass of about 70-72 kDa in sodium dodecylsulphate polyacrylamide gel electrophoresis (SDS PAGE) [28]. The P2X7 subunit comprises two transmembrane-spanning helices about 24 amino acids long, a short intracellular N-terminal domain and an extended intracellular carboxy-terminal chain, critical for several unique P2X7R functions. The bulky extracellular domain is glycosylated and harbors 10 cysteines [20]. The *P2RX7* gene is highly polymorphic, with more than 1500 human single nucleotide polymorphisms (SNPs) identified [29,30]. It comprises 13 coding exons and is located on the long arm of chromosome 12 at 12q24.31. P2X7R gating opens a transmembrane, cation-selective, channel that promotes Ca^2+^ and Na^+^ influx and K^+^ efflux [26], but may also induce the formation of a large non-selective pore (the “macropore”), allowing unrestricted transmembrane passage of large molecules of a molecular mass up to 900 Da [20,26,31]. Macropore formation causes a dramatic disruption of intracellular ion homeostasis and may trigger cell death by apoptosis, necrosis, or pyroptosis [32]. On the other hand, compelling evidence shows that tonic activation of the P2X7R by low autocrinally- or paracrinally-released eATP, may also support a trophic effect [33]. Therefore, this dual function confers an unusual plasticity to the P2X7R as a cytotoxic or alternatively growth-promoting receptor. This yin/yang P2X7R activity is also reflected in the intracellular energy metabolism, where a low level of P2X7R stimulation supports mitochondrial oxidative phosphorylation [33], whereas overstimulation precipitates a “mitochondrial catastrophe” highlighted by Ca^2+^ flooding of the mitochondrial matrix, uncoupling of the respiratory chain, and mitochondrial fragmentation [34].

The main claim to fame of the P2X7R is as a pro-inflammatory receptor [35] as it is the most potent plasma membrane receptor coupled to activation of the NACHT, LRR, and PYD domain containing protein 3 (NLRP3), inflammasome activation, recruitment of adaptor apoptosis-associated speck-like protein containing a C-terminal caspase recruitment domain (ASC), and cleavage of pro-caspase-1 into its activated form, i.e., caspase-1 [35]. The end result of this chain of events is caspase-1-mediated cleavage of pro-IL-1β and pro-IL-18 into mature IL-1β and IL-18, respectively, and their release into the extracellular space [26,35,36]. P2X7R activation is also associated to the release of other cytokines and chemokines, such as IL-6, TNF-α, CCL2, IL-8, CCL3, and CXCL2 [35,37]. While the main trigger of P2X7R-dependent NLRP3 inflammasome stimulation is a drop in intracellular K^+^, the parallel increase in the cytoplasmic Ca^2+^ and Na^+^ concentrations on one hand activates a number of additional pathways such as nuclear factor kappa B (NF-kB), nuclear factor of activated T-cells (NFAT), phosphoinositide 3-kinases/serine/threonine-protein kinase (PI3K/Akt), and on the other causes down modulation of glycogen synthase kinase 3 beta (GSK3β) [26,32]. All of these unique P2X7R features are attracting increasing attention by pharmaceutical companies for the development of novel anti-inflammatory drugs.

## 3. Type 2 Diabetes Mellitus (T2DM)

In physiological conditions, an increase in blood glucose levels triggers glucose uptake by pancreatic β-cells through facilitated diffusion. This process is mainly mediated by the GLUT-1 glucose transporter 1 in humans, and by GLUT-2 in mice [38,39,40]. Intracellular glucose catabolism increases the ATP/ADP ratio, which leads to the closure of ATP-sensitive potassium channels (K_ATP_), plasma membrane depolarization, cytosolic Ca^2+^ increase and exocytosis of insulin-containing granules [40,41]. The incidence and prevalence of T2DM have been steadily increasing due to obesity, sedentary lifestyles and high-calorie diets (modifiable risks), as well as to population aging, ethnicity and genetic predisposition [40,42,43]. It is estimated that T2DM accounts for over 90% of total DM cases [44]. The main pathogenic factor in T2DM is the inability of insulin-sensitive tissues to respond to insulin, and in the progressive establishment of a condition of defective insulin secretion from pancreatic cells, which eventually leads to a stably elevated blood glucose level (hyperglycemia) [40,45]. The main organs affected in T2DM are pancreas, liver, skeletal muscle, kidneys, brain, small intestine, and adipose tissue [40,46]. Obesity and reduced physical activity are relevant risk factors for T2DM because adipose tissue inflammation impairs adipose tissue function, insulin sensitivity, and glucose metabolism [40]. In most obese people, insulin resistance is paralleled by increased blood insulin levels, likely a compensatory factor. However, this initial hyperinsulinemia is followed by a steady decline in β-cell function and an increased β-cell apoptosis, leading to decreased β-cell mass [47,48,49]. Obesity is associated with chronic low-grade systemic inflammation (metabolic inflammation) as witnessed by increased blood concentration of TNF-α, C-reactive protein (CRP) and IL-1β [40,50,51]. Moreover, the proinflammatory cytokines are also involved in promoting insulin resistance in insulin-sensitive organs, resulting in T2DM development. In the adipose tissue, macrophages are polarized to a proinflammatory phenotype, secreting large proinflammatory cytokine quantities that exacerbate inflammation and insulin resistance. In addition, HFD promotes the formation of reactive oxygen species (ROS), leading to an impairment of mitochondrial function characterized by ATP overproduction in the absence of a parallel increase in cellular energy demand. This dysfunction is referred to as “mitochondrial overheating” [52,53]. Furthermore, high iATP levels, on one hand, promote the phosphorylation of several kinases such as the inhibitor of nuclear factor kappa-B kinase subunit beta (IKKβ), Jun N-terminal kinase (JNK) and extracellular signal-regulated kinases (ERK), that in turn promote transcriptional expression of inflammatory cytokines [50]. On the other hand, they also inhibit the serine/threonine kinase AMP-activated protein kinase (AMPK) complex [54], which decreases GLUT-4 activity and reduces insulin-stimulated glucose uptake [54]. 

Pro-inflammatory cytokines, e.g., IL-6, TNF-α, and IL-1β, have a leading role in the establishment of insulin resistance via activation of serine kinases, such as protein kinase C (PKC), mitogen-activated protein kinases (MAPK) and the inhibitor of NF-kB kinase complex beta (IKKβ). These kinases inhibit activation of the insulin signaling cascade at the insulin receptor substrate (IRS) level, and drive IRS degradation [55,56,57,58]. T2DM is also characterized by impaired insulin secretion due to β-cell loss [59,60], an additional dire effect of the enhanced secretion of pro-inflammatory cytokines (IL-6, TNF-α, and IL-1β) [61,62,63,64]. IL-6 inhibits transcription of IRS-1 and GLUT-4 genes, TNF-α down-regulates the GLUT-4 gene, and IL-1β reduces IRS-1 expression, inhibits GLUT-4 translocation to the plasma membrane, and overall lowers insulin-stimulated glucose uptake [65,66]. Inhibition of IRS-1 expression by IL-1β occurs via an ERK-dependent transcriptional mechanism (inhibition of IRS-1 mRNA expression), and an ERK-independent post transcriptional pathway [65,66]. It has been shown that IL-1β-stimulated nitric oxide (NO) production from β-cells can be toxic to the β-cells themselves and inhibit iron-containing mitochondrial enzymes, thus causing an impairment of oxidative phosphorylation, a reduction of iATP and decreased insulin secretion [67,68]. Thus, in T2DM the mitochondria may be injured by two different (and opposite) mechanisms: excessive ATP production in the absence of a matching energy requirement (“mitochondrial overheating”), or outright impairment of oxidative phosphorylation.

Energy metabolism and the mitochondria are gaining increasing importance for our understanding of the pathogenesis of T2DM. Skeletal muscle mitochondria of T2DM subjects are smaller in comparison with lean subjects, and individuals with insulin resistance show impaired mitochondrial activity compared to those without insulin resistance [69,70]. Finally, crucial contribution of IL-1β to T2DM pathogenesis is demonstrated by the reduction of blood glucose levels and the improvement β-cells function in T2DM and T1DM patients treated with the interleukin-1 receptor antagonist (IL-1Ra) [49,71,72,73,74,75,76,77,78,79].

## 4. The P2X7R in β-Cells Pathophysiology and Type 2 Diabetes Mellitus

**Figure 1:**
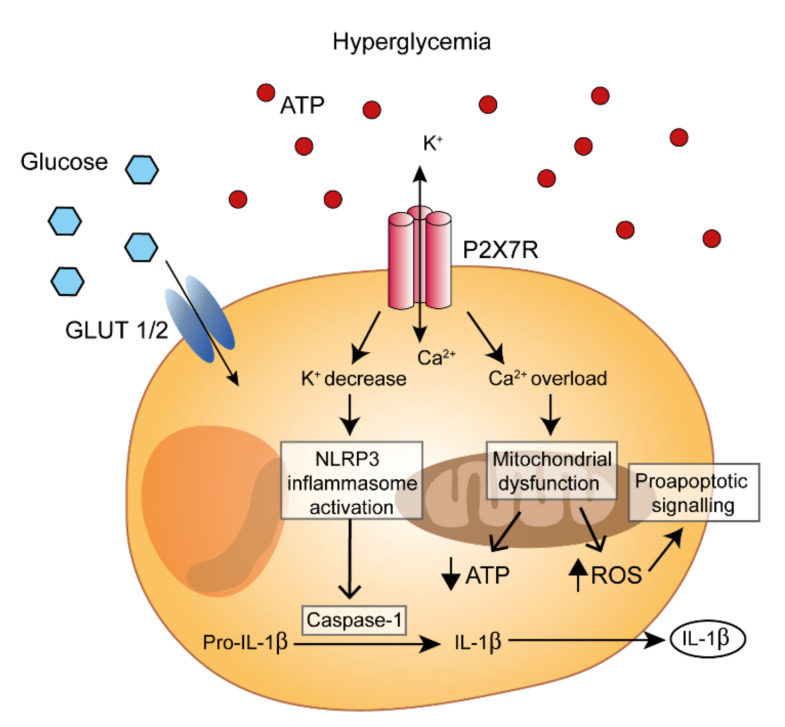
P2X7R modulation of β-cell pro-inflammatory functions. Hyperglycemia promotes eATP release and P2X7R-dependent K^+^ efflux and Ca^2+^ influx into the β-cells. Ca^2+^ overload promotes mitochondrial dysfunction, reactive oxygen species (ROS) generation, inhibition of ATP synthesis, and eventually triggers apoptosis. Moreover, intracellular K^+^ decrease induces the assembly of the NLRP3 inflammasome, promotes IL-1β release and further accelerates β-cell apoptosis.

ATP has a dual role in the regulation of insulin secretion: as an intracellular modulator of K_ATP_ channels and as an extracellular signaling molecule [80,81,82,83]. Multiple sources of eATP are known: nerve terminals, inflammatory cells, and the pancreatic β-cells themselves. Pancreatic β-cells concentrate ATP into insulin-containing granules via the vesicular nucleotide transporter (VNUT), therefore stimulation by hyperglycemia will cause Ca^2+^-dependent co-release of ATP with insulin [15,16]. Additional pathways for ATP release by β-cells are the P2X7R and pannexin-1 pathways [81]. In β-cells, P2X7R and pannexin-1 are upregulated by high glucose levels thus generating an amplifying loop of the effects of hyperglycemia [81]. Another relevant mechanism for ATP release under stressful conditions is cell injury or death (eATP is a well-known damage-associated molecular pattern (DAMP)), which may be relevant to the inflamed pancreas. Stimulation of P2 receptors (P2Rs) by eATP triggers a large increase in the intracellular Ca^2+^ concentration, which further potentiates exocytosis of secretory granules and therefore insulin release [81,84,85,86]. However, stimulation by eATP seems to be self-limiting, as while low eATP levels promote, excessive eATP levels inhibit insulin release irrespective of the glucose concentration, suggesting that eATP might function as an autocrine regulator of β-cell function [87,88]. Based on the different sensitivity to eATP, it is postulated that stimulation of insulin secretion at low eATP concentrations is mediated by P2YRs, while inhibition at high eATP concentrations is mainly due to ectonucleotidase-mediated adenosine accumulation [88]. The main enzymes responsible for the generation of extracellular adenosine, NTPDase-1/CD39 and ecto-5′nucleotidase/CD73, and P1 adenosine receptors are expressed in pancreatic islets [1,82,89,90]. High eATP levels will also promote P2X7R activation. Although the P2X7R plays an important role in the physiology of β-cells, supporting cell survival and potentiation of insulin secretion [49,81,91], it may also promote β-cell damage, either directly or indirectly via release of IL-1β. In fact, P2X7R stimulation is a very potent stimulus for NLRP3 inflammasome activation and IL-1β processing and release (Figure 1).

**Figure 2:**
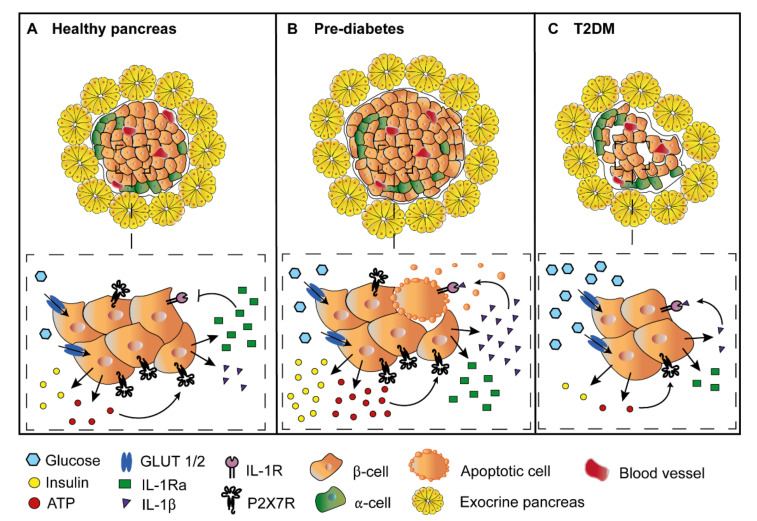
Role of the P2X7R in the healthy pancreas, in pre-diabetic conditions and overt T2DM. (**A**) In healthy subjects, low grade P2X7R stimulation contributes to β-cell homeostasis by keeping a careful balance of IL-1β and IL-1Ra release, and at the same time providing a trophic stimulus that supports β-cell proliferation. (**B**) In pre-diabetic conditions, hyperglycemia triggers insulin and ATP release, which stimulates the P2X7R, that in turn promotes enhanced release of both IL-1β and IL-1Ra. IL-1Ra release prevents, at least in part, the deleterious effects of IL-1β on β-cells. However, continued β-cell stimulation by sustained hyperglycemia, on one hand leads to a progressive exhaustion of insulin secretion, and on the other hand, causes a large increase in the intra-islet eATP concentration, causing an unchecked release of IL-1β. In this context, IL-1Ra cannot anymore protect β-cells from IL-1β effects. The combined action of IL-1β and eATP then promotes β-cell apoptosis and the shrinkage of Langerhans islet mass. (**C**) In overt T2DM, β-cell number is reduced, insulin secretion is impaired, and P2X7R expression by β-cells is downmodulated.

Interestingly, P2X7R activation also promotes IL-1Ra release, possibly via an increase in intracellular Ca^2+^ levels [49,76,77]. P2X7R expression is increased in pancreas of obese individuals and circulating levels of IL-1Ra are increased in subjects with obesity and insulin resistance. On the contrary, in overt T2DM, P2X7R expression and circulating IL-1Ra levels are reduced. This might depend on the upregulation of the P2X7R during the initial phases of hyperglycemia, which promotes P2X7R-stimulated release of IL-1β and IL-1Ra, followed by, at later stages (overt diabetes), β-cell death and decreased IL-Ra plasma levels [49,79,92,93,94,95,96] (Figure 2).

Tonic stimulation of the P2X7R might support β-cell proliferation [91], therefore it can be anticipated that its downmodulation or deletion will also be detrimental for β-cell function and overall glycemic homeostasis. This hypothesis is supported by the observation that the P451L P2X7R hypofunctional polymorphic variant in rodents leads to an impaired glucose tolerance and reduced insulin responsivity [97], and by a recent study showing that in T2DM individuals presence of the hyperfunctional variant A348T increased insulin release and promoted an overall improvement of β-cell function [98].

In view of the pivotal role of inflammation in T2DM, understanding the role of the P2X7R in immune cells is of fundamental importance. Previous work showed that CD14^+^, CD4^+^ and CD19^+^ T cell subpopulations from T2DM patients express higher P2X7R levels when compared to healthy patients, and CD39^+^/CD19^+^ cells were associated with HbA1c and increased fasting plasma glucose levels [99]. Furthermore, in T2DM patients, P2X7R expression in human peripheral blood monocytes correlates with TNF-α, IL-1β, and CRP plasma levels [100]. Therefore, it is likely that P2X7R activation enhances the release of pro-inflammatory cytokines, thus perpetuating inflammation [99,101]. In addition, inflammation is a major mechanism of cell and tissue damage, as shown by the participation of the P2X7R in several T2DM complications, such as diabetic kidney disease, diabetic retinopathy and diabetic neuropathy [91,102]. P2X7R-mediated modulation of pancreatic stellate cells (PSCs) by eATP/P2X7R might be an additional complicating factor. Activated PSCs release large amounts of extracellular matrix components that promote islet fibrosis and β-cell dysfunction. The P2X7R is reported to promote PSC proliferation and activation and, when overstimulated, PSC death [103]. Pancreases isolated from P2X7-KO mice show a lower number of PSCs versus wild-type mice, supporting a trophic function for this receptor. PSC might be another crucial player in the overall process of diabetogenic P2X7R activation.

## 5. Conclusions

T2DM is a chronic disease characterized by subclinical inflammation likely responsible for abnormal insulin secretion and peripheral insulin resistance. Inflammation is associated with increased levels of eATP, a biochemical subversion of the extracellular environment, that in the pancreas might drastically affect insulin secretion and peripherally impair insulin sensitivity. Thus, P2X7R targeting might be an appealing option for T2DM therapy.
